# Identifying Patients With Inflammatory Bowel Disease on Twitter and Learning From Their Personal Experience: Retrospective Cohort Study

**DOI:** 10.2196/29186

**Published:** 2022-08-02

**Authors:** Maya Stemmer, Yisrael Parmet, Gilad Ravid

**Affiliations:** 1 Department of Industrial Engineering and Management Ben-Gurion University of the Negev Beer-Sheva Israel

**Keywords:** patient identification, inflammatory bowel disease, IBD, user classification, Twitter, natural language processing, NLP, sentiment analysis

## Abstract

**Background:**

Patients use social media as an alternative information source, where they share information and provide social support. Although large amounts of health-related data are posted on Twitter and other social networking platforms each day, research using social media data to understand chronic conditions and patients’ lifestyles is limited.

**Objective:**

In this study, we contributed to closing this gap by providing a framework for identifying patients with inflammatory bowel disease (IBD) on Twitter and learning from their personal experiences. We enabled the analysis of patients’ tweets by building a classifier of Twitter users that distinguishes patients from other entities. This study aimed to uncover the potential of using Twitter data to promote the well-being of patients with IBD by relying on the wisdom of the crowd to identify healthy lifestyles. We sought to leverage posts describing patients’ daily activities and their influence on their well-being to characterize lifestyle-related treatments.

**Methods:**

In the first stage of the study, a machine learning method combining social network analysis and natural language processing was used to automatically classify users as patients or not. We considered 3 types of features: the user’s behavior on Twitter, the content of the user’s tweets, and the social structure of the user’s network. We compared the performances of several classification algorithms within 2 classification approaches. One classified each tweet and deduced the user’s class from their tweet-level classification. The other aggregated tweet-level features to user-level features and classified the users themselves. Different classification algorithms were examined and compared using 4 measures: precision, recall, F1 score, and the area under the receiver operating characteristic curve. In the second stage, a classifier from the first stage was used to collect patients' tweets describing the different lifestyles patients adopt to deal with their disease. Using IBM Watson Service for entity sentiment analysis, we calculated the average sentiment of 420 lifestyle-related words that patients with IBD use when describing their daily routine.

**Results:**

Both classification approaches showed promising results. Although the precision rates were slightly higher for the tweet-level approach, the recall and area under the receiver operating characteristic curve of the user-level approach were significantly better. Sentiment analysis of tweets written by patients with IBD identified frequently mentioned lifestyles and their influence on patients’ well-being. The findings reinforced what is known about suitable nutrition for IBD as several foods known to cause inflammation were pointed out in negative sentiment, whereas relaxing activities and anti-inflammatory foods surfaced in a positive context.

**Conclusions:**

This study suggests a pipeline for identifying patients with IBD on Twitter and collecting their tweets to analyze the experimental knowledge they share. These methods can be adapted to other diseases and enhance medical research on chronic conditions.

## Introduction

### Background

Social networking sites and web-based communities have served as alternative information sources for patients in recent years. Patients everywhere use social media to share health and treatment information, learn from each other’s experiences, and provide social support. Mining these informative conversations may shed some light on patients’ ways of life and support research on chronic conditions.

In recent years, text mining and social network analysis have been used to detect mentions of health on Twitter [1,2] or to track the spread of the COVID-19 pandemic and symptoms [3-5]. Regarding chronic conditions, previous research has focused on analyzing patients’ tweets and uncovering their Twitter community [6-10]. Although a relatively large amount of research has been dedicated to diabetes or cancer, research on inflammatory bowel disease (IBD) is only just starting to consolidate.

IBD is a chronic inflammatory condition of the digestive system characterized by flares and remission states. The 2 primary diseases identified with IBD, Crohn disease and ulcerative colitis, are usually diagnosed in young patients (in the age range of 15-30 years). The incidence of IBD is rapidly increasing, and it has evolved into a global disease [11-14].

There are no medications or surgical procedures that can cure IBD. Treatment options can only help with symptoms, and they affect each patient differently. They involve prescription drugs and lifestyle-related solutions such as diets and therapies. Symptoms include abdominal pain, diarrhea, and fatigue, and severe cases may result in hospitalization or surgical interventions [15,16]. As chronic bowel diseases, both Crohn disease and ulcerative colitis require day-to-day care for drug consumption and special nutrition.

Patients describe IBD as an embarrassing disease that causes the immediate disruption of daily activities. They experience difficulties in adjusting to the changes it entails and consider themselves different from their peers. As IBD is characterized by frequent bowel movements, people do not hasten to share their disease with others [15,17-19]. According to patients with IBD, part of the embarrassment can be attributed to a lack of public awareness. Outsiders cannot see that a person’s stomach hurts or that their bowels are scarred. The disease is invisible, and others might doubt that it exists [20,21].

The embarrassment caused by IBD and the need to confide in people with similar experiences help explain the creation of IBD-related communities on Twitter. By overcoming space and distance, Twitter users form a community that disregards physical boundaries or immobility. A sense of common ground can help break down barriers and enable conversation, increasing a person’s willingness to share [22,23]. It may be easier to consult with other patients who can relate and better understand the situation based on personal experience. One can identify more closely with user stories similar to one’s own and embrace their advice more easily [24]. When people disclose health information on Twitter, they expose themselves to a large variety of opinions and reduce the uncertainty about their disease [25].

Owing to the nature of IBD and its influence on the digestive system, patients with IBD are forced to deal with their disease daily, adhere to strict dietary regimens, and maintain a calm routine. Changes in nutrition or physical activity, which are currently tested by trial and error, result in a long and excruciating process for the patients. We can learn from their personal experiences and provide an additional foundation for existing medical knowledge of the disease by collecting and analyzing patients’ social media data. Complementary recommendations based on the wisdom of the crowd can ease patients’ lives and shorten the process of finding the right lifestyle for them.

### Objective and Contribution

This study aimed to uncover the potential of using Twitter data to promote the well-being of patients with IBD by collecting and analyzing the personal experiences they share about their disease. We suggested a framework for identifying patients with IBD on Twitter and examining the content they share regarding their disease. We started by building a user classifier that distinguishes patients from other entities who talk about IBD on Twitter and then used the classifier to collect patients’ tweets and explore the lifestyle-related treatments they undergo to cope with their disease.

This study focused on creating a pipeline for using Twitter data for identifying patients with IBD and exploring the information they share. Although each part of this study can be extended by trying other classification methods or enriching the analysis of the patients’ tweets, this study shows the potential of using Twitter data to enhance medical knowledge of IBD. We showed that patients can be identified on Twitter based on their communication even using classic, simple classification algorithms. We compared the performances of 2 different approaches for user classification—a single instance (SI) learning approach and a multiple instance (MI) learning approach—and showed the benefits of using the latter. The preliminary analysis in the second part of this study showed that it is possible to derive health-related insights from self-reported tweets by patients.

Using the suggested framework to identify more patients and collect more of their data could uncover their sentiments toward the treatments they try or explore other aspects of the disease, such as its influence on patients’ quality of life. The framework is also feasibly extended to other chronic conditions. It can be used to compare discussion patterns of patients with IBD with those of the general population or of patients with other chronic conditions.

### Related Work

#### Twitter and Health

The study of social media in the context of health and well-being continues to position Twitter as a new medium for disseminating health-related information. Health-related tweets range from a simple toothache to more severe and chronic diseases such as diabetes, asthma, or cancer [9,10,26,27]. Patients with amyotrophic lateral sclerosis use Twitter as a means of communication, and local health departments in the United States use Twitter to educate and disseminate information related to diabetes [28,29]. Even a sensitive disease such as HIV is discussed on Twitter [30-32]. Communication patterns regarding who tweets about what and why vary by disease [26].

Twitter is a powerful tool for disseminating health information and an accessible platform for patients needing immediate social support or relief. It provides a collaborative environment for health-related conversations where patients with chronic illnesses share their health status daily. They use Twitter to exchange knowledge about lifestyle implications or better understand a medical procedure. Through Twitter, they can easily and conveniently reach a large audience and various opinions [33].

In total, 2 previous studies have presented models for detecting personal health mentions on Twitter and shown promising, scalable results [1,2]. However, their goal differs from ours as they considered all tweets that discussed a specific person’s health condition as positive. In our study, we sought to identify patients with a specific disease. We not only classified tweets written by patients but also classified the users themselves.

#### Communication Patterns on Twitter

Different types of users communicate differently on Twitter. They connect differently with others, have different tweeting habits, and differ in style and linguistic content. Studying the conversational connections between Twitter users and text mining their tweets can help classify users based on their characteristics and identify different types of users [34-38].

Private individuals reflect mainly on their personal experiences or sentiments and tend to engage with others. They are both frequently mentioned and frequently mentioning other users. By contrast, organizations often point to external information sources via URLs and are not that active at connecting with others. They are frequently mentioned in tweets, perhaps as sources of information, but are much less inclined to mention other users [39,40].

By analyzing a user’s screen name (ie, the username of their Twitter account) or their biography (ie, their Twitter user description), one can determine whether the user is an ordinary individual or an organization and reveal latent user properties [41,42].

Our study relies on those previous findings and constructs classification features that help differentiate patients with IBD from other users who tweet about the disease. We adapted and extended previous methods to cope with the different task of identifying patients with IBD on Twitter.

#### Twitter and IBD

Exploring the entities that engage in IBD-related discussions on Twitter reveals that patients with IBD are the most common type of users who talk about IBD on Twitter [43,44]. Patients with IBD use Twitter for sharing personal experiences and seeking social support. They exchange thoughts about symptoms and medications and recommend treatments to one another [45,46]. By sharing their life experiences with the disease on Twitter, patients fight disease invisibility and raise public awareness of IBD [47].

Perez et al [48] explored the IBD community on Twitter and identified the types of users who talk about the disease and the key topics they discuss. They categorized users based on their Twitter profiles by analyzing their screen names and biographies. In our study, we investigated a large set of classification features and suggested a model to detect patients with IBD on Twitter based on the way they communicate and the content they share.

Patients with IBD tend to be more emotional and negative than patients with other chronic conditions [49]. They usually express a negative sentiment when they talk about the disease and its symptoms but positively address the diets and drugs that help manage them [48]. Patients who engage in tweets offering social support are more likely to post positive tweets [50].

Unlike previous research related to patients’ sentiments on Twitter [48-50], we focused our research on entity sentiment rather than the sentiment of the entire tweet. By analyzing patients’ sentiments toward specific keywords related to nutrition and fitness, we uncovered the sentiments of certain lifestyles that influence the disease.

## Methods

### Overview

This study was conducted in 2 main stages. In the Patient Identification section, we built a user classifier that distinguishes patients from other entities who talk about IBD on Twitter. We considered three types of classification features: (1) features extracted from the user’s activity on Twitter, (2) the content of the user’s tweets, and (3) the social structure of the user’s network. We compared the performances of several classification algorithms within 2 classification approaches: one that starts by classifying tweets separately and then deduces the user’s class from their tweet-level classification and one that starts by aggregating tweet-level features to user-level features and then classifies the users themselves.

In the Analyzing Patients’ Tweets section covering the second stage of the study, we derived insights regarding IBD from the personal experiences that patients share on Twitter. We collected lifestyle-related tweets by querying the Twitter application programming interface (API) for special keywords related to nutrition or fitness. We then filtered their authors using a classifier from the first stage of the study to obtain a collection of tweets where patients with IBD describe the different diets and physical activities they adopt to deal with their disease. We identified frequently mentioned lifestyles and used IBM Watson Service for entity sentiment analysis to assess their effectiveness.

**Figure 1:**
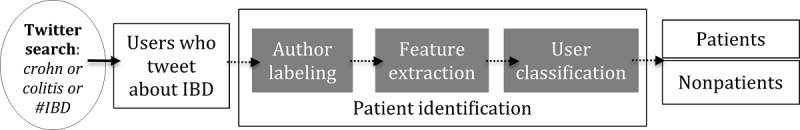
The general workflow of the first stage of the study: building a classifier of Twitter users for identifying patients with inflammatory bowel disease (IBD).

**Figure 2:**
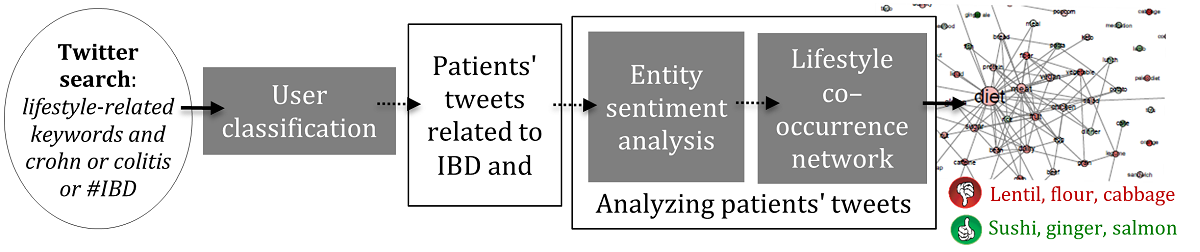
The general workflow of the second stage of the study: using the classification from the first stage for analyzing patients' tweets. IBD: inflammatory bowel disease.

Figures 1 and 2 describe the general flow of the 2 main stages of the study. Figure 1 describes how we used Twitter data to classify users and identify patients with IBD. Figure 2 demonstrates how we used the classification to analyze patients' tweets.

### Patient Identification

#### Data Collection and Preparation

We used the Twitter Search API to collect 10 days of IBD-related tweets (from February 11, 2018, at noon to February 21, 2018, at noon). We used the OR operator to search for at least one of 3 keywords: *crohn*, *colitis,* and *#IBD*. The abbreviation IBD was searched as a hashtag to avoid news-related tweets by the Investor’s Business Daily Editorials account, which is usually marked with *IBD*. We limited the search to tweets written in English and collected 2045 tweets.

The 722 authors of the collected tweets were then manually classified as patients (1) or not (0). In total, 3 different annotators, the authors of this paper (MS, YP, and GR), did the labeling process and labeled the users based on their tweets. Each user received a tag of 1 if they had at least one tweet revealing their illness and a tag of 0 otherwise (ie, if none of their tweets suggested that they were patients with IBD).

Regarding 655 users (n=181, 27.6% patients and n=474, 72.4% other users), the annotators were in complete agreement, and their labels were set. To settle the dispute regarding the other 9.3% (67/722) of the users, the annotators challenged their tweet-based decisions by considering the users’ screen names and biographies and reviewing their timelines if necessary. Considering the new data, of the 67 remaining users, 45 (67%) were classified as patients after explicitly mentioning their illness in their biographies or timelines. A total of 12% (8/67) talked about others who were sick, and the annotators unanimously agreed that they were not patients with IBD themselves. Regarding the remaining 21% (14/67) users, the annotators did not reach a consensus and, therefore, the users were omitted from the data set. The labeling process ended with a collection of 708 tagged users: 226 (31.9%) patients and 482 (68.1%) nonpatients.

To train the tweet-level classifiers, we had to annotate the tweets manually as well. We addressed the tweets collected in the original search query (in February 2018) and excluded retweets (RTs) from the collection. As the purpose was to identify patients, we were not interested in reshared content and only considered the user’s tendency to RT as a behavioral classification feature. After excluding RTs and the 14 users for whom we did not reach an annotation consensus, we were left with 1687 tweets. To consider the users’ biographies as we did when annotating users, we added each biography as another *tweet* by its author. A total of 83.5% (591/708) of the users had nonempty biographies, and the process resulted in a collection of 2278 *tweets*.

During the annotation process, we wanted to determine whether a certain tweet revealed that the user was a patient with IBD. Tweets that unambiguously implied that their authors were patients with IBD received a tag of 1, and all others received a tag of 0. As we had already annotated the users, all 1638 tweets written by nonpatient users automatically received a tag of 0. The 3 annotators (MS, YP, and GR) then manually classified all the tweets written by patients. A total of 346 tweets were unanimously classified as 1, and 288 tweets were unanimously classified as 0. The annotators did not reach a consensus on 6 tweets (written by 6 different users), and they were excluded from the collection. All 6 users had at least one more tweet and, therefore, none of them were excluded entirely from our data set. Finally, we reached a collection of 2272 tweets, of which 346 (15.23%) explicitly revealed their authors’ illness.

To enrich our data, we collected another week of tweets (from June 10, 2018, at noon to June 17, 2018, at noon) for each tagged user, this time without additional filtering. In the months that had passed, 6.6% (47/708) of the users had been either suspended by Twitter or changed their accounts to private, and their data were no longer available for collection. The additional week was collected for the other 93.4% (661/708) of the users, and the process resulted in a data set of 82,884 tweets overall written by 194 patients and 467 nonpatients. We excluded the same 47 users from the tweet data set as well, and the final data set contained 2204 tweets, with 325 (14.75%) positive tweets.

#### MI Learning Approach

Traditional classification problems are supervised learning problems in which one receives a collection of individually labeled instances and tries to predict the class label for new instances. MI learning, by contrast, is a supervised learning approach in which each learning example is a *bag of instances* associated with 1 label, and the task is to predict the labels for unseen bags [51].

Previous research related to identifying health mentions on Twitter has relied on traditional supervised learning to determine whether a *tweet* discusses a health condition [1,2]. However, we wished to determine whether *patients* can be identified on Twitter and not examine the tweets separately. Our unique task and the unbalanced structure of our data were compatible with an MI learning approach—we had 661 users and a different number of tweets posted by each of them. Positive tags (patients) were determined collectively by finding at least one piece of evidence that the user had IBD; negative tags (nonpatients) meant that all the user’s evidence suggested otherwise or, rather, was not sufficient for a positive tag.

We used the metadata-based MI approach and extracted a vector of metadata for each bag (user) that was not related to any specific instance (tweet) [52]. The Classification Features section explains how we applied feature engineering techniques to generate features that characterize the users themselves and not just their tweets.

To assess the effectiveness of using this collective approach, we compared the results of 5 standard classification algorithms in both user- and tweet-level classification, as explained in detail in the Classification Models section.

#### Classification Features

##### Overview

Rao et al [38] and Pennacchiotti and Popescu [36,37] showed that Twitter users’ demographics and political views could be distinguished by considering 3 types of user classification features: behavioral features (features extracted from the user’s activity on Twitter), linguistic features (features extracted from the content of the user’s tweets), and social structure features (features describing the user’s social network). We followed their work and adapted these types to our different domains of distinguishing patients with IBD from others who talk about the disease. We also integrated MI learning into our classification setting, which was not part of their research. We constructed a set of classification features for each feature type, as explained in detail in the following sections and summarized in Table 1.

**Table 1:** Summary of classification features and their types.

User classification feature, feature level, and features			Type
**Behavioral features**			
	**Tweet-level features**		
		Tweet counter	Integer
		Retweet counter	Integer
		Retweet to tweet ratio	Float (0 to 1)
		IBD^a^ flag	Binary
		User-level IBD ratio	Float (0 to 1)
		Crohn flag	Binary
		User-level Crohn ratio	Float (0 to 1)
		Colitis flag	Binary
		User-level colitis ratio	Float (0 to 1)
	**User-level features**		
		Tweet counter	Integer
		Retweet counter	Integer
		Retweet to tweet ratio	Float (0 to 1)
		IBD counter	Integer
		Bio-IBD flag	Binary
		IBD ratio	Float (0 to 1)
		Crohn counter	Integer
		Bio-Crohn flag	Binary
		Crohn ratio	Float (0 to 1)
		Colitis counter	Integer
		Bio-colitis flag	Binary
		Colitis ratio	Float (0 to 1)
**Linguistic features**			
	**Tweet-level features**		
		Emoji counter	Integer
		Interjection counter	Integer
		Profanity counter	Integer
		Mention counter	Integer
		Hashtag counter	Integer
		URL flag	Binary
		First-person flag	Binary
		Number of words	Integer
		Number of characters	Integer
		Polarity	Float (−1 to 1)
		Positive polarity flag (1 if polarity >0, else 0)	Binary
		Negative polarity flag (1 if polarity <0, else 0)	Binary
		Subjectivity	Float (0 to 1)
		LDA^b^ topic distribution (document=tweet)	20×float (0 to 1)
	**User-level features**		
		Emoji sum	Integer
		Emoji average	Float
		Bio-emoji counter	Integer
		Interjection sum	Integer
		Interjection average	Float
		Bio-interjection counter	Integer
		Profanity sum	Integer
		Profanity average	Float
		Bio-profanity counter	Integer
		Mention sum	Integer
		Mention average	Float
		Bio-mention counter	Integer
		Hashtag sum	Integer
		Hashtag average	Float
		Bio-hashtag counter	Integer
		URL sum	Integer
		URL average	Float (0 to 1)
		Bio-URL flag	Binary
		First-person sum	Integer
		First-person average	Float (0 to 1)
		Bio–first-person flag	Binary
		Word average	Float
		Bio-number of words	Integer
		Character average	Float
		Bio-number of characters	Integer
		Bio-polarity	Float (−1 to 1)
		Positive polarity sum	Integer
		Positive polarity average	Float (0 to 1)
		Negative polarity sum	Integer
		Negative polarity average	Float (0 to 1)
		Subjectivity average	Float (0 to 1)
		Bio-subjectivity	Float (0 to 1)
		LDA topic distribution (document=all the user’s tweets)	20×float (0 to 1)
**Social structure features**			
	**Tweet-level features**		
		User-level log in-degree	Float
		User-level log out-degree	Float
		User-level closeness	Float (0 to 1)
	**User-level features**		
		Log in-degree	Float
		Log out-degree	Float
		Closeness	Float (0 to 1)

^a^IBD: inflammatory bowel disease.

^b^LDA: latent Dirichlet allocation.

##### Behavioral Features

Features of this type were designed to capture users’ activity on Twitter: How often do they tweet? Do they write new content or mainly RT others? Furthermore, how often do they refer to IBD? We counted the number of tweets and RTs in our data set and calculated the RT ratio for each user. We counted the number of times they used one of our keywords in their tweets to account for the frequency with which they addressed IBD. Aggregated features for user-level classification were also copied to all the users’ tweets to enrich the tweet-level classification.

##### Linguistic Features

The second class of features is derived from the users’ linguistic style on Twitter: Do they write in first-person voice? Do they tend to use emoticons or add a reference to an external source via URL? We used 2 types of linguistic features. On the basis of previous research [36-38] and our data’s nature, we extracted several features from the text that we believed would help the classification.

Acknowledging that individuals and organizations communicate differently on Twitter [35,39], we searched for specific characteristics that could distinguish private persons from businesses and help identify patients. We checked specific characteristics for each tweet in our data: Was there use of emojis, interjections, or profanities? Was it written in the first person? Did it point to an external source via URL? Did it contain Twitter special characters indicating mentions (@) or hashtags (#)? We used a Python (Python Software Foundation) library called *TextBlob* to add sentiment-related features such as the text’s polarity and subjectivity. The length of the tweets and the number of words they contained were also considered. The Python library *emoji* was used to detect emojis within the text. A part-of-speech identifier from the library *nltk* was used to indicate the use of first person and identify interjections. On the basis of the Python library *profanity*, we established a list of swear words that we searched for in the text. We had to adjust the list to the special domain of IBD as words related to metabolism were not necessarily swear words.

We started with tweet-level features, which were later grouped by user to represent personal writing style. To reflect the way a user expresses themselves on Twitter, we excluded RTs from the aggregation. The number of tweets in which the URL was used, for example, was counted on the original tweets only. As the users’ biographies were considered as tweets in the tweet-level classifiers, we added the linguistic features that were extracted from the biographies as bio-features in the user-level classifiers.

In natural language processing, there are several methods to obtain a vector representation of text. One of the more well-known and well-researched techniques is the Bayesian probabilistic model of text documents called latent Dirichlet allocation (LDA). LDA is a topic modeling technique used for discovering the abstract *topics* that occur in a collection of documents [53].

We used LDA to represent text in both tweet- and user-level classification features. In tweet-level features, each tweet was considered a document, and the representations were obtained per tweet. For user-level features, all tweets by the same author were consolidated into 1 document to obtain representations per user. All the features used unigram and bigram representations of the text after data cleaning. The text cleaning process included converting to lower case, removing punctuation and stop words, and normalizing links and other special signs to standard representations.

##### Social Structure Features

The last type of feature we addressed represented the users’ social connections on Twitter. We used the Twitter API to collect each user’s followers and followees. For each user, we kept the number of followers they had (out-degree in the sense of influence) and the number of followees they had (in-degree) and scaled the results using a logarithmic scale. We also computed the closeness centrality measure for each user. Aggregated features for user-level classification were also copied to all the users’ tweets to enrich the tweet-level classification.

#### Classification Models

Aiming to distinguish between patients with IBD and other users who tweet about IBD, we compared the performances of several classification algorithms within 2 classification approaches: the SI learning approach, which starts by classifying tweets separately and then deduces the user’s class from their tweet-level classification, and the metadata-based MI learning approach, which starts by aggregating tweet-level features to user-level features and then classifies the users themselves.

The metadata-based MI approach starts by transforming the data from MI to SI, and then a standard SI algorithm can be applied to the transformed problem [54,55]. To achieve the users’ characterization for the MI approach, we applied arithmetic sum and average to the tweet-level features and obtained aggregated features per user (refer to the Classification Features section for more details). Note that this process may cause some information loss [56].

For both approaches, we tested 5 standard and well-known algorithms for binary classification tasks such as ours: AdaBoost, gradient boosting classifier, linear support vector machine, logistic regression, and random forest. All the algorithms were applied from the scikit-learn (sklearn) package in Python [57].

#### Experiment

We split our data set by users into training and test sets (approximately 80%-20%). The training set had 155 patients and 377 nonpatients, and the test set had 39 patients and 90 nonpatients; thus, the sets maintained the ratio between the groups.

In the tweet-level classification, the split into training and test sets was performed based on the split of the users—tweets by users belonging to the training set were ascribed to the tweet training set, whereas tweets by users belonging to the test set were ascribed to the tweet test set. As a result, the tweet training set contained 263 positive tweets and 1586 negative tweets, whereas the test set contained 62 positive tweets and 293 negative tweets.

We started with a hyperparameter optimization for all algorithms using a 5-fold cross-validation over the training data in both approaches. The values tested for each algorithm and parameter can be found in Multimedia Appendix 1.

In total, 4 common metrics were used to evaluate the models: precision, recall, F_1_ score, and the area under the receiver operating characteristic curve (ROC AUC). All 4 metrics were calculated over the positive class that was of interest to us. In our setting, precision depicts the probability that a positive prediction is indeed a patient, recall depicts the classifier’s ability to retrieve patients, and the F_1_ score combines the 2. ROC AUC considers the recall of both classes and measures the ability of the model to retrieve patients without collecting a lot of unwanted other users.

To select the best algorithm variant, we used a 10-fold cross-validation technique for a reliable evaluation of the prediction power. In this process, we randomly divided the training set into 10 equal-sized parts; then, we iteratively performed the training on 9 parts and evaluated the model on the part that was left out. We repeated this iteration 10 times, leaving out a different part each time. In addition, we repeated the 10-fold cross-validation process 10 times with different seed initializations to vary the random split. The performance metrics were computed each time, and the results presented in the Results section show the average across these 100 iterations.

In the user-level classification, we obtained all 4 metrics during the classification process using the sklearn package in Python. However, in the tweet-level classification, another aggregation stage was needed before obtaining the metrics directly from the sklearn package—the process returned the predictions for each tweet (whether it was written by a patient), and we had to infer the users’ predictions by aggregating the predictions given to their tweets. As in the manual annotation process, if all the user’s tweets received a prediction of 0, the user was considered a nonpatient and received a negative prediction. Alternatively, if the user had at least one positive prediction, they were considered a patient and received a positive prediction. We then used the sklearn package to compute the user-level metrics based on the users’ predictions that we obtained and their true labels.

Finally, we trained the models from each approach (MI and SI) on the entire training set and evaluated their predictions on the test set. We used built-in sklearn methods for feature importance to investigate the contribution of each feature to both logistic regression and random forest algorithms. The absolute value of the coefficient represents the feature importance for logistic regression.

### Analyzing Patients’ Tweets

#### A Corpus of Lifestyle-Related Tweets

The next aim of this study was to obtain a collection of tweets in which patients describe the lifestyle-related treatments they have tried and their symptoms. By filtering and merging different web-based databases [58,59], we established a list of 420 words that are types of food or physical activities (ie, lifestyle-related words; the full list can be found in Multimedia Appendix 2). The Twitter Premium API was used to search for all tweets that mentioned IBD (containing at least one of the 3 keywords described in the Data Collection and Preparation section: *crohn*, *colitis*, and *#IBD*) and at least one of the 420 lifestyle-related words. To build the search query, we used the OR operator within the IBD keywords and the lifestyle-related words and then connected the 2 groups using the AND operator.

We searched for relevant tweets from January 1, 2019, to September 30, 2019. We excluded RTs and duplicated tweets from the search and limited the search to tweets written in English. The search resulted in 20,136 unique tweets containing new content written by 8519 different users.

We used the classifier from the first part of the study on the new data we gathered to classify the tweets as patients’ tweets and user tweets. We needed to recreate the classification features for the new set of 8519 users. As we did in the first stage, we collected another week of tweets for all the users from October 1, 2019, to October 7, 2019, without keyword filtering and including RTs. A total of 39.52% (3367/8519) of the users were private, suspended, or otherwise unavailable. The process resulted in a data set of 5152 users who authored 402,843 tweets overall.

We constructed all the classification features described in the Classification Features section on the new data except for the closeness centrality. Obtaining this feature was costly and time-consuming as it was the only feature that required collecting all followers and followees for each user and building their Twitter network. As it was not one of the 10 most helpful classification features, we decided to omit it.

We then used the MI random forest model we trained in the first stage (refer to the Classification Models section for more details) to classify the users and identify patients. A total of 45.79% (2359/5152) of the users were classified as patients, and they authored 4160 of the original tweets containing our keywords. We performed a simple text cleaning of those tweets by removing all screen names (identified by the @ character) and URLs and continued our analysis with the 4160 clean tweets.

#### Sentiment Analysis of Lifestyle-Related Words

The Natural Language Understanding (NLU) module by IBM Cloud [60] was used to apply category classification and keyword extraction to each of our tweets. The category classification feature aims to identify the theme of the text. Given a text, the NLU module provides a list of possible categories and subcategories and their corresponding likelihoods. The keyword extraction feature recognizes words and phrases of high importance within the text and calculates their sentiments. Given a text, the NLU module returns a list of keywords and their corresponding sentiments represented as scores on the closed interval of −1 to 1: −1 for extremely negative sentiment and 1 for extremely positive sentiment. A score of 0 means that the keyword was mentioned in a neutral context. The *TextBlob* library used for sentiment analysis in the Linguistic Features section only enables full-text sentiment analysis and does not support entity-level sentiment analysis. Although it was free and easy to use, it did not suit our new task and, therefore, we chose to replace it with the NLU module.

The goal was to identify the lifestyle-related treatments that patients undergo to manage their disease and determine their sentiments toward them. Hence, we focused our analysis on keywords related to health and nutrition. We grouped all tweets that were categorized by the NLU module as related to *health and fitness* (2080 tweets), *food and drink* (1568 tweets), or *religion and spirituality* (15 tweets). Overall, 3663 tweets were selected for keyword sentiment analysis. We gathered all the keywords that appeared in our predefined list of lifestyle-related words and their corresponding sentiments within each tweet. In total, 3 examples of this process are presented in Table 2. Notice how, in the second example, the first word of the original tweet (marked with the @ symbol) is a screen name and was therefore removed in the cleaning process.

**Table 2:** Three examples of category classification and keyword sentiment extraction after text cleaning.

Number	Original text	Text after cleaning	Category classification	Keyword sentiment
1	Spinach is an inflammatory food with a lot of sulfur. Ban that too. (I noticed my Crohn’s tended to flare around spinach season.)	Spinach is an inflammatory food with a lot of sulfur. Ban that too. (I noticed my Crohn’s tended to flare around spinach season.)	Food and drink	Spinach: −0.63
2	@bottomline_ibd great poll. I do have the odd binge, but IBD has changed what I can drink. No more red wine or ale 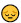	great poll. I do have the odd binge, but IBD has changed what I can drink. No more red wine or ale 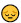	Food and drink	Red wine: −0.83; ale: −0.83
3	I am living proof that yoga can help #uchicagoibd #studiothree #yoga #ibd	I am living proof that yoga can help #uchicagoibd #studiothree #yoga #ibd	Religion and spirituality	Yoga: 0.69

To examine the effectiveness of each lifestyle-related phrase (lifestyle, in short) and to assess its overall sentiment, we aggregated the results by lifestyle and calculated the following statistics: the total number of times the lifestyle appeared in all tweets, the number of times it appeared in a positive (or negative) context, the positive to negative ratio of the number of appearances (odds), and the mean sentiment of the lifestyle.

We used the statistics to build a co-occurrence network that visualized the connections between lifestyles and their mean sentiments. The different lifestyles were the nodes, and an arc connected 2 lifestyles if they appeared in the same tweet. The more times they appeared together, the stronger the connection between the lifestyles was. Therefore, the resulting network was undirected and weighted by the number of times the lifestyles co-occurred. The purpose was to identify helpful lifestyles (frequently mentioned in a positive context) and lifestyles that it is better to avoid (frequently mentioned in a negative context) and examine whether certain lifestyles tend to be implemented together.

The network was obtained using *Gephi* software (GNU General Public License) for network analysis and visualization. Each node was colored on a scale from green to red based on the mean sentiment of the lifestyle it represented, with green being very positive and red being very negative. The sizing of the nodes reflected the number of times the lifestyles were mentioned in the tweet database: the more times they appeared, the larger their nodes were. The thickness of each arc represented the number of times the 2 lifestyles it connected co-occurred: the thicker the arc, the more times the 2 lifestyles appeared together. To avoid obtaining an overdense network, we only considered the nodes of lifestyles mentioned at least five times in our database. We included arcs between lifestyles that co-occurred at least four times. The process resulted in 144 lifestyles presented in the network and sorted in a table by mean sentiment.

### Ethical Note

The collection and analysis of Twitter data may entail ethical challenges that should be addressed and handled properly. Twitter data are public and available for research via Twitter APIs. By accepting Twitter’s Terms of Service and Privacy Policy, Twitter users acknowledge that their tweets can be viewed instantly worldwide and that their information may be collected by third parties [61]. Nonetheless, social media studies have revealed that users on Twitter feel as if they are engaged in a private conversation with their followees and followers [62,63]. Although they are generally not concerned with their posts being used for research purposes, they expect anonymity in publication and to be asked for their consent before publication.

Obtaining informed consent from all the users who *participate* in research on Twitter data may be unfeasible. Data sets are likely to be large and involve many authors [61-63]. Individually seeking consent from all 722 users in our study would be labor-intensive or impossible as some might be unreachable. Moreover, providing total anonymity to users while directly quoting their content is not practical; tweets are easily searchable, leaving their authors vulnerable to identification.

To adhere to ethical norms and maintain user privacy, we only published aggregated results that do not reveal the specific users. The 3 examples containing direct quotes from tweets (in Table 2) are presented in this study after obtaining informed consent from their authors.

## Results

### Patient Identification

Table 3 shows the 10-fold cross-validation and test results for the 2 classification approaches: SI classifying tweets and MI classifying users. The table shows the results of the 4 metrics for all 5 classification algorithms.

**Table 3:** The 10-fold cross-validation and test results for the single instance (SI) and multiple instance (MI) classifications.

Algorithm and metric			SI tweet-level classification					MI user-level classification		
			10-fold		Test		10-fold			Test
**AdaBoost**										
	Precision	0.6775		0.7241		0.6151			0.5902	
	Recall	0.6297		0.5385		0.7284			0.9231	
	F_1_ score	0.6525		0.6176		0.6542			0.7200	
	ROC AUC^a^	0.7532		0.7248		0.8469			0.8226	
**Gradient boosting classifier**										
	Precision	0.7416		0.6471		0.6668			0.6735	
	Recall	0.6465		0.5641		0.6778			0.8462	
	F_1_ score	0.6906		0.6027		0.6711			0.7500	
	ROC AUC	0.7768		0.7154		0.8658			0.8342	
**Linear SVM^b^**										
	Precision	0.7249		0.6667		0.6648			0.5814	
	Recall	0.6832		0.7179		0.6398			0.6410	
	F_1_ score	0.7034		0.6914		0.6472			0.6098	
	ROC AUC	0.7883		0.7812		0.8463			0.7205	
**Logistic regression**										
	Precision	0.7405		0.6333		0.6594			0.6250	
	Recall	0.6335		0.4872		0.6358			0.6410	
	F_1_ score	0.6829		0.5507		0.6423			0.6329	
	ROC AUC	0.7712		0.6825		0.8473			0.7372	
**Random forest**										
	Precision	0.7676		0.7333		0.6721			0.6444	
	Recall	0.4355		0.2821		0.6646			0.7436	
	F_1_ score	0.5555		0.4074		0.6595			0.6905	
	ROC AUC	0.6906		0.6188		0.8722			0.7829	

^a^ROC AUC: area under the receiver operating characteristic curve.

^b^SVM: support vector machine.

**Figure 3:**
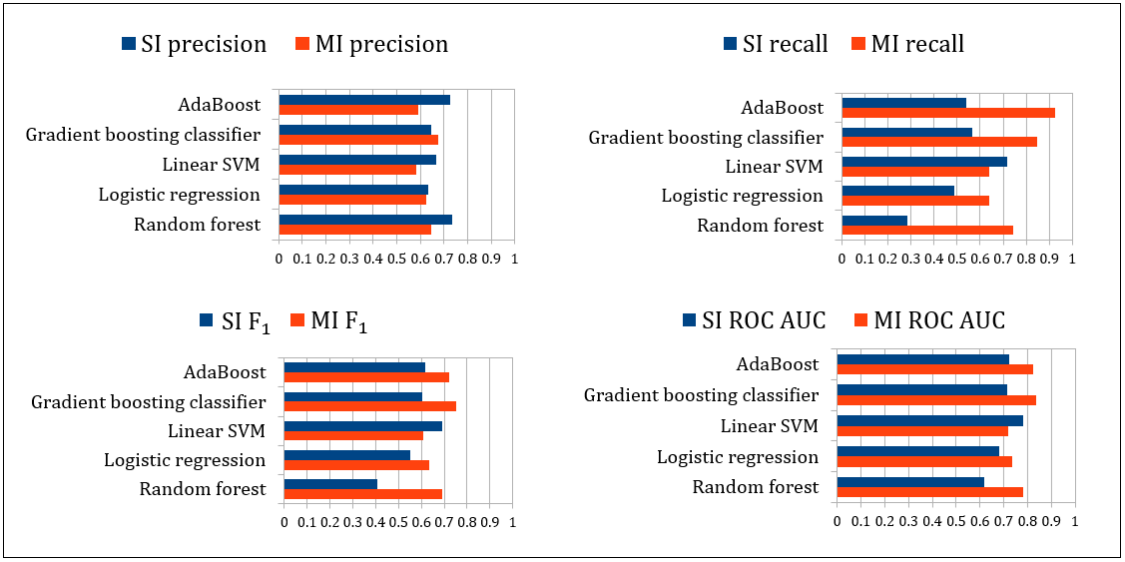
Test result comparison between the 2 classification approaches. MI: multiple instance; ROC AUC: area under the receiver operating characteristic curve; SI: single instance; SVM: support vector machine.

Both approaches showed satisfactory classification results for the patient classes. Although the precision rates were slightly higher for the SI approach, the recall index of the MI approach was better, and the results for the ROC AUC measure were consistently higher in the MI approach. Figure 3 shows the differences among the 4 measures within the test set results.

Investigating the contribution of each feature to both the logistic regression and random forest algorithms showed the importance of the use of first-person voice. In both classification approaches and algorithms, the most important feature was the use of the first person, which had a significant advantage over the other features. The first-person flag was the best feature of the SI approach, and its average was the best feature of the MI approach. Another dominant feature was the use of profanities as it was one of the most significant features in both approaches and algorithms.

The analysis also highlighted the importance of the LDA features derived from the text. The second-best feature of the SI approach was LDA topic 11 for both the logistic regression and random forest algorithms. This was the only topic that did not contain IBD-related words. The fourth and fifth most important topics of the MI approach were identical for both algorithms—LDA topics 17 and 9, respectively. The LDA topics that were created over the training data for each approach can be found in Multimedia Appendix 3.

### Analyzing Patients’ Tweets

In the second stage of the study, a network of connections between lifestyles was built and visualized. The obtained network describing the relationships between the different lifestyles can be found in Multimedia Appendix 4.

The most frequent word in our database was *diet*, encapsulating all the nutritional adjustments that patients undergo to manage their disease. Specific diets such as *paleo*, *vegetarian*, or *liquid* diets also surfaced and in a negative context.

It is interesting to note that the negative and positive lifestyles revealed by the analysis were in line with what is known about suitable nutrition for IBD. Among the most negative lifestyles (by mean sentiment), we found *alcohol, milk, spicy, cabbage, flour, lentil,* and *orange juice*, all known to cause inflammation and irritate the stomach. Among the most positive lifestyles (by mean sentiment), we found activity-related lifestyles such as *fitness* or *yoga* and healing foods such as *salmon*, *ginger*, and *garlic*. The most positive lifestyle turned out to be *sushi*, which usually contains anti-inflammatory ingredients such as *salmon* or *tuna*, *seaweed*, and *rice*. Table 4 presents the 20 most positive and 20 most negative lifestyle-related words sorted by mean sentiment.

**Table 4:** The 20 most positive and 20 most negative lifestyles sorted by mean sentiment.

Rank	Keyword	Count	Sentiment, mean (SD)	Count of positive	Count of negative	Odds
1	Sushi	9	0.466 (0.814)	7	2	3.500
2	Ginger ale	5	0.407 (0.597)	3	1	3.000
3	Salmon	7	0.344 (0.691)	4	3	1.333
4	Cherry	10	0.33 (0.696)	6	2	3.000
5	Breakfast	29	0.28 (0.75)	19	9	2.111
6	Garlic	8	0.244 (0.671)	4	2	2.000
7	Bagel	5	0.224 (0.633)	3	1	3.000
8	Almond	9	0.193 (0.668)	6	3	2.000
9	Yogurt	14	0.189 (0.688)	7	3	2.333
10	Yoga	15	0.186 (0.693)	7	5	1.400
11	Ham	5	0.184 (0.535)	2	1	2.000
12	Biscuit	13	0.172 (0.75)	8	5	1.600
13	Spinach	6	0.171 (0.76)	4	2	2.000
14	Vegan cheese	5	0.164 (0.92)	3	2	1.500
15	Lamb	5	0.14 (0.861)	3	2	1.500
16	Cake	26	0.13 (0.752)	16	9	1.778
17	Fitness	19	0.114 (0.728)	9	6	1.500
18	Ginger	17	0.112 (0.724)	8	7	1.143
19	Tomato	10	0.089 (0.608)	5	3	1.667
20	Cafe	7	0.081 (0.783)	3	3	1.000
125	Fodmap	12	−0.501 (0.573)	2	9	0.222
126	Cocktail	5	−0.51 (0.769)	1	4	0.250
127	Fiber	63	−0.512 (0.547)	7	47	0.149
128	Spicy	37	−0.514 (0.572)	7	28	0.250
129	Vegetable	49	−0.533 (0.529)	6	39	0.154
130	Corn	28	−0.534 (0.487)	2	22	0.091
131	Alcohol	64	−0.545 (0.545)	9	51	0.176
132	Milkshake	5	−0.556 (0.811)	1	4	0.250
133	Milk	44	−0.565 (0.5)	4	35	0.114
134	Vegetarian diet	10	−0.567 (0.409)	1	8	0.125
135	Snack	10	−0.573 (0.568)	2	8	0.250
136	Fig	5	−0.578 (0.621)	1	4	0.250
137	Turkey	10	−0.608 (0.626)	2	8	0.250
138	Yeast	16	−0.624 (0.391)	1	13	0.077
139	Orange	7	−0.638 (0.449)	0	5	0.000
140	Beverage	7	−0.661 (0.616)	1	6	0.167
141	Cabbage	8	−0.675 (0.19)	0	8	0.000
142	Orange juice	5	−0.682 (0.385)	0	4	0.000
143	Flour	6	−0.785 (0.211)	0	6	0.000
144	Lentil	6	−0.785 (0.188)	0	6	0.000

## Discussion

### Principal Findings

This study presents a workflow for identifying patients with IBD on Twitter and exploring their tweets. The aim was to identify patients with IBD based on the way they communicate on Twitter and to learn from the personal experiences they share.

In the first stage of the study, a classifier of Twitter users designed to distinguish patients with IBD from other users was constructed and evaluated. Classification features combining social data and text analysis were extracted from the users’ activity on Twitter, their social connections, and the content of their tweets. Various classification algorithms were considered, and 4 evaluation measures were calculated for each of them. The encouraging results shown in the previous section helped convince us that patients with IBD can be identified on Twitter based on such features.

Classification results from both the SI and MI approaches show that patients with IBD differ in the way they communicate on Twitter from other users who tweet about the disease. They talk in the first person more often and use more profanities in their tweets. These gaps, which can be explained by the fact that patients are private individuals whereas nonpatients also include organizations and voluntary associations that communicate in a much more formal manner, helped distinguish patients from other entities in the different classification models we tried in this study.

Our analysis differs from previous research regarding user classification on Twitter [36-38] in 2 aspects. Conceptually, we investigate a different domain and try to identify patients on Twitter. Practically, we compare the results from the user-level classification with a tweet-level classification.

In the second stage of the study, tweets of patients with IBD were collected to investigate the different lifestyles they implemented to deal with their disease and assess these lifestyles’ effectiveness. Unlike previous research on patients’ sentiments on Twitter [48-50], we focused our research on entity sentiment for specific words rather than the entire tweet’s sentiment. We suggested a novel approach by considering entity sentiment analysis to obtain patients’ sentiments toward the different nutrition and fitness-based solutions they try. These findings were in line with what is known about IBD as several foods known to cause inflammation were pointed out in a negative sentiment, whereas relaxing activities and anti-inflammatory foods surfaced in a positive context.

This study suggests that there is room for collaboration between physicians and engineers regarding understanding chronic diseases. Owing to the chronic nature of the disease and the fact that it involves bowel movements, patients with IBD are compelled to follow special nutrition and maintain a calm routine. By collecting and analyzing patients’ personal experiences on social media, we can monitor patients’ lifestyles and support medical knowledge of IBD. We can identify and assess complementary treatments to diets and physical activity and maybe ease patients’ processes of finding the right treatments for them. Although such analysis should not strive to replace physicians or draw conclusions of a clinical nature, it may provide complementary recommendations for healthy lifestyles based on the wisdom of the crowd.

### Limitations and Future Work

#### Overview

The focus of this study was on showing the potential of identifying patients with IBD on Twitter and learning from their tweets. This study emphasized the entire process, and we did not perfect each part separately. As this section explains, each part can be improved by trying different methods and enriching the analysis.

#### Patient Identification

The classifier developed in the first stage of this study uses 1-level, binary classification to separate patients with IBD from other users who tweet about the disease. Some of its features distinguish organizations from individuals in general and do not necessarily detect patients, such as the use of the first person in the tweet. Therefore, our nonpatient class is heterogeneous and somewhat ambiguous, containing both organizations that significantly differ from patients in their communication patterns and healthy individuals who differ from patients in a more refined manner. Even during the manual labeling process, all 14 users excluded from the data set owing to classification disagreements were individuals talking in the first-person voice.

A possible direction for future work would be to try a 2-step classification: separating persons from organizations and continuing by searching for patients among these individuals. It can improve the robustness of some of the features by overcoming the heterogeneity of the nonpatient class in our model. Alternatively, we could try replacing the binary classification with a multinomial one that will capture not only organizations and patients but also individuals who talk about the disease and maybe mention other patients but are not sick themselves.

During the construction of the network-based features, we only collected immediate connections on Twitter (ie, the followers and followees of each patient). The sampling method resulted in basic network features that mainly included degree measures. We encourage future research to consider more interesting network features such as other centrality measures or structures. Such enhancement will require collecting at least one more level of connections (eg, followees of followees) to understand network patterns better.

Finally, the classifier uses standard classification algorithms and did not try current state-of-the-art learning techniques based on neural networks. Text representation using word embeddings, where words are mapped to vectors of real numbers in a predefined vector space [64,65], is also worth examining.

#### Analyzing Patients’ Tweets

The NLU module by IBM Cloud was used in this study for entity sentiment analysis as a proof of concept. We did not evaluate its results or compare them with similar tools available in the market, such as the Natural Language API by Google Cloud. Future research should consider performing similar analyses with different natural language processing tools and comparing their results. Even training designated algorithms on data from lifestyle-related tweets such as those used in this study can benefit the analysis.

Overall, the results for the second part are preliminary, and much more can be done to understand what patients with IBD are talking about on Twitter. For example, by characterizing treatment options and patients’ sentiments toward them, one can derive recommendations for a healthy lifestyle based on the wisdom of the crowd. Thoroughly exploring outliers, such as the 4 positive mentions of milk as opposed to the 35 negative ones, can reveal new information regarding the disease that has not yet been covered in the literature.

### Conclusions

In the era of personalized medicine and patient-centered care, it is important to derive insights that reflect the patients’ perspectives as manifested in social media. Although the time between physician appointments can be lengthy, messages on social media are being posted each day, and patients constantly use them to exchange inputs and recommendations.

This study provides a potential pipeline for identifying patients with chronic illnesses on Twitter and collecting their tweets to analyze the experimental knowledge they share on the web. The method presented in this study was applied to IBD and can also help explore other medical conditions. The classifier for IBD-related entities can be adapted to identify other patients with chronic illnesses. The analysis of patients’ tweets can benefit research on other chronic conditions with similar characteristics. With conditions such as celiac disease or diabetes, which involve strict dietary guidelines, one can better understand patients’ difficulties with adherence to their new lifestyles. When considering diseases that cause embarrassment, such as HIV, one can learn more about the constant struggle of patients living with the disease.

Therefore, the contribution of this study is 2-fold: it provides an analytical contribution to the fields of text mining and social media and a practical contribution by better understanding chronic conditions and promoting a healthy lifestyle for patients with chronic illnesses.

## Appendix

Multimedia Appendix 1 Parameter optimization for classification algorithms.

Multimedia Appendix 2 A list of 420 lifestyle-related words.

Multimedia Appendix 3 Latent Dirichlet allocation topics created over the training data for each classification approach.

Multimedia Appendix 4 A network of relationships between lifestyle-related words.

## Abbreviations

API: application programming interface
IBD: inflammatory bowel disease
LDA: latent Dirichlet allocation
MI: multiple instance
NLU: Natural Language Understanding
ROC AUC: area under the receiver operating characteristic curve
RT: retweet
SI: single instance
