# Supplementary material for: Identifying Patients With Inflammatory Bowel Disease on Twitter and Learning From Their Personal Experience: Retrospective Cohort Study
Source: J Med Internet Res. 2022 Aug 2;24(8):e29186. doi: 10.2196/29186 (PMC9382547; doi:10.2196/29186)
Supplement: Multimedia Appendix 1 [file jmir_v24i8e29186_app1.docx]

## Multimedia Appendix 1

Parameter optimization for classification algorithms.

|  | Parameter | Values |
| --- | --- | --- |
|  |  |  |
| **AdaBoost** |  |  |
|  | Number of estimators | 1, 2, 10, 50, 100 |
|  | Algorithm | SAMME.R, SAMME |
| **Gradient Boosting Classifier** |  |  |
|  | Number of estimators | 20, 40, 60, 80, 100 |
|  | Max depth | 3, 7, 11, 15 |
|  | Min sample split | 2, 4, 6, 8, 10 |
| **Linear SVM** |  |  |
|  | C | 0.1, 0.5, 1, 10 |
| **Logistic Regression** |  |  |
|  | C | 0.001, 0.01, 0.1, 1, 10, 100, 1000 |
|  | Penalty | L1, l2 |
| **Random Forest** |  |  |
|  | Number of estimators | 50, 100, 150, 200 |
|  | Max features | Auto, sqrt, log2 |
|  | Max tree depth | 3, 4, 5, 6, 7 |
|  | Criterion | Gini, entropy |
