# Supplementary material for: Identifying Patients With Inflammatory Bowel Disease on Twitter and Learning From Their Personal Experience: Retrospective Cohort Study
Source: J Med Internet Res. 2022 Aug 2;24(8):e29186. doi: 10.2196/29186 (PMC9382547; doi:10.2196/29186)
Supplement: Multimedia Appendix 2 [file jmir_v24i8e29186_app2.docx]

## Multimedia Appendix 2

List of 420 lifestyle-related keywords, organized by category.

| Word Id | Category | Product |
| --- | --- | --- |
|  |  |  |
| 1 | alcohol | alcohol |
| 2 | alcohol | beer |
| 3 | alcohol | brandy |
| 4 | alcohol | cocktail |
| 5 | alcohol | daiquiri |
| 6 | alcohol | gin |
| 7 | alcohol | liqueur |
| 8 | alcohol | margarita |
| 9 | alcohol | martini |
| 10 | alcohol | mimosa |
| 11 | alcohol | mojito |
| 12 | alcohol | rum |
| 13 | alcohol | sangria |
| 14 | alcohol | scotch |
| 15 | alcohol | tequila |
| 16 | alcohol | vodka |
| 17 | alcohol | whiskey |
| 18 | alcohol | wine |
| 19 | cheese | brie |
| 20 | cheese | camembert |
| 21 | cheese | cheddar |
| 22 | cheese | cottage |
| 23 | cheese | feta |
| 24 | cheese | gouda |
| 25 | cheese | gruyere |
| 26 | cheese | mozzarella |
| 27 | cheese | parmesan |
| 28 | cheese | provolone |
| 29 | cheese | ricotta |
| 30 | cheese | roquefort |
| 31 | coffee | cafe |
| 32 | coffee | caffeine |
| 33 | coffee | cappuccino |
| 34 | coffee | coffee |
| 35 | coffee | espresso |
| 36 | coffee | latte |
| 37 | coffee | macchiato |
| 38 | coffee | mocha |
| 39 | cooking form | baked |
| 40 | cooking form | boiled |
| 41 | cooking form | broiled |
| 42 | cooking form | cooked |
| 43 | cooking form | fried |
| 44 | cooking form | grilled |
| 45 | cooking form | roasted |
| 46 | dairy | butter |
| 47 | dairy | buttermilk |
| 48 | dairy | cheese |
| 49 | dairy | cream |
| 50 | dairy | custard |
| 51 | dairy | dairy |
| 52 | dairy | eggnog |
| 53 | dairy | gelato |
| 54 | dairy | kefir |
| 55 | dairy | milk |
| 56 | dairy | milkshake |
| 57 | dairy | yogurt |
| 58 | drink | beverage |
| 59 | drink | cocoa |
| 60 | drink | cola |
| 61 | drink | drink |
| 62 | drink | juice |
| 63 | drink | lemonade |
| 64 | drink | nectar |
| 65 | drink | punch |
| 66 | drink | smoothie |
| 67 | drink | soda |
| 68 | drink | tea |
| 69 | drink | water |
| 70 | eggs | egg |
| 71 | eggs | french toast |
| 72 | eggs | frittata |
| 73 | eggs | omelette |
| 74 | eggs | quiche |
| 75 | eggs | yolk |
| 76 | fish | anchovy |
| 77 | fish | clam |
| 78 | fish | codfish |
| 79 | fish | crab |
| 80 | fish | fish |
| 81 | fish | herring |
| 82 | fish | lobster |
| 83 | fish | mackerel |
| 84 | fish | mullet |
| 85 | fish | oyster |
| 86 | fish | salmon |
| 87 | fish | sardine |
| 88 | fish | seafood |
| 89 | fish | shrimp |
| 90 | fish | sushi |
| 91 | fish | tuna |
| 92 | fitness | activity |
| 93 | fitness | aerobics |
| 94 | fitness | fitness |
| 95 | fitness | gym |
| 96 | fitness | jogging |
| 97 | fitness | meditate |
| 98 | fitness | meditation |
| 99 | fitness | mindfulness |
| 100 | fitness | pilates |
| 101 | fitness | swim |
| 102 | fitness | swimming |
| 103 | fitness | workout |
| 104 | fitness | yoga |
| 105 | fruit | acai |
| 106 | fruit | apple |
| 107 | fruit | apricot |
| 108 | fruit | avocado |
| 109 | fruit | banana |
| 110 | fruit | bearberry |
| 111 | fruit | berry |
| 112 | fruit | bilberry |
| 113 | fruit | blackberry |
| 114 | fruit | blueberry |
| 115 | fruit | boysenberry |
| 116 | fruit | cactus |
| 117 | fruit | cantaloupe |
| 118 | fruit | carambola |
| 119 | fruit | cherry |
| 120 | fruit | citron |
| 121 | fruit | citrus |
| 122 | fruit | clementine |
| 123 | fruit | coconut |
| 124 | fruit | cranberry |
| 125 | fruit | currant |
| 126 | fruit | dried date |
| 127 | fruit | eucalyptus |
| 128 | fruit | feijoa |
| 129 | fruit | fig |
| 130 | fruit | fruit |
| 131 | fruit | goji |
| 132 | fruit | gooseberry |
| 133 | fruit | grape |
| 134 | fruit | grapefruit |
| 135 | fruit | guava |
| 136 | fruit | huckleberry |
| 137 | fruit | kiwi |
| 138 | fruit | lemon |
| 139 | fruit | lime |
| 140 | fruit | loganberry |
| 141 | fruit | loquat |
| 142 | fruit | lychee |
| 143 | fruit | mandarin |
| 144 | fruit | mango |
| 145 | fruit | melon |
| 146 | fruit | nectarine |
| 147 | fruit | orange |
| 148 | fruit | papaya |
| 149 | fruit | passion fruit |
| 150 | fruit | peach |
| 151 | fruit | pear |
| 152 | fruit | persimmon |
| 153 | fruit | pineapple |
| 154 | fruit | pitaya |
| 155 | fruit | plum |
| 156 | fruit | pomegranate |
| 157 | fruit | pomelo |
| 158 | fruit | prune |
| 159 | fruit | quince |
| 160 | fruit | raisin |
| 161 | fruit | raspberry |
| 162 | fruit | rosehip |
| 163 | fruit | starfruit |
| 164 | fruit | strawberry |
| 165 | fruit | tangelo |
| 166 | fruit | tangerine |
| 167 | fruit | watermelon |
| 168 | fruit | youngberry |
| 169 | general | breakfast |
| 170 | general | cornmeal |
| 171 | general | dinner |
| 172 | general | eat |
| 173 | general | flour |
| 174 | general | fondue |
| 175 | general | food |
| 176 | general | gelatin |
| 177 | general | ice |
| 178 | general | lunch |
| 179 | general | protein |
| 180 | general | ratatouille |
| 181 | general | rice |
| 182 | general | salad |
| 183 | general | souffle |
| 184 | general | soup |
| 185 | general | stew |
| 186 | general | supper |
| 187 | general | tofu |
| 188 | legume/ seeds | bran |
| 189 | legume/ seeds | cereal |
| 190 | legume/ seeds | chickpeas |
| 191 | legume/ seeds | grain |
| 192 | legume/ seeds | granola |
| 193 | legume/ seeds | grits |
| 194 | legume/ seeds | legume |
| 195 | legume/ seeds | lentil |
| 196 | legume/ seeds | oat |
| 197 | legume/ seeds | oatmeal |
| 198 | legume/ seeds | porridge |
| 199 | legume/ seeds | quaker |
| 200 | legume/ seeds | quinoa |
| 201 | legume/ seeds | seeds |
| 202 | legume/ seeds | sesame |
| 203 | meat | bacon |
| 204 | meat | beef |
| 205 | meat | bologna |
| 206 | meat | bratwurst |
| 207 | meat | burger |
| 208 | meat | chicken |
| 209 | meat | chorizo |
| 210 | meat | frankfurter |
| 211 | meat | ham |
| 212 | meat | hamburger |
| 213 | meat | hot dog |
| 214 | meat | lamb |
| 215 | meat | meat |
| 216 | meat | meatball |
| 217 | meat | mortadella |
| 218 | meat | pastrami |
| 219 | meat | pepperoni |
| 220 | meat | pork |
| 221 | meat | salami |
| 222 | meat | sausage |
| 223 | meat | sirloin |
| 224 | meat | steak |
| 225 | meat | turkey |
| 226 | meat | veal |
| 227 | nut | almond |
| 228 | nut | cashew |
| 229 | nut | chestnut |
| 230 | nut | hazelnut |
| 231 | nut | nut |
| 232 | nut | peanut |
| 233 | nut | pecan |
| 234 | nut | pistachio |
| 235 | nut | walnut |
| 236 | nutrition | diet |
| 237 | nutrition | gluten |
| 238 | nutrition | nutrition |
| 239 | nutrition | organic |
| 240 | nutrition | paleo |
| 241 | nutrition | vegan |
| 242 | nutrition | vegetarian |
| 243 | pasta | cannelloni |
| 244 | pasta | gnocchi |
| 245 | pasta | lasagna |
| 246 | pasta | linguini |
| 247 | pasta | macaroni |
| 248 | pasta | manicotti |
| 249 | pasta | noodle |
| 250 | pasta | pad thai |
| 251 | pasta | pappardelle |
| 252 | pasta | pasta |
| 253 | pasta | penne |
| 254 | pasta | ravioli |
| 255 | pasta | rigatoni |
| 256 | pasta | spaghetti |
| 257 | pasta | tortellini |
| 258 | pastry | bagel |
| 259 | pastry | baguette |
| 260 | pastry | biscotti |
| 261 | pastry | biscuit |
| 262 | pastry | bread |
| 263 | pastry | brioche |
| 264 | pastry | bun |
| 265 | pastry | burrito |
| 266 | pastry | cracker |
| 267 | pastry | crepe |
| 268 | pastry | croissant |
| 269 | pastry | croutons |
| 270 | pastry | dumpling |
| 271 | pastry | empanada |
| 272 | pastry | enchilada |
| 273 | pastry | fajita |
| 274 | pastry | loaf |
| 275 | pastry | matzo |
| 276 | pastry | muffin |
| 277 | pastry | pastry |
| 278 | pastry | patty |
| 279 | pastry | pie |
| 280 | pastry | pizza |
| 281 | pastry | pretzel |
| 282 | pastry | quesadilla |
| 283 | pastry | sandwich |
| 284 | pastry | taco |
| 285 | pastry | toast |
| 286 | pastry | tortilla |
| 287 | pastry | waffle |
| 288 | pastry | weaht |
| 289 | pastry | yeast |
| 290 | sauce/ spread | canola |
| 291 | sauce/ spread | catsup |
| 292 | sauce/ spread | dressing |
| 293 | sauce/ spread | gravy |
| 294 | sauce/ spread | hollandaise |
| 295 | sauce/ spread | horseradish |
| 296 | sauce/ spread | ketchup |
| 297 | sauce/ spread | margarine |
| 298 | sauce/ spread | mayonnaise |
| 299 | sauce/ spread | molasses |
| 300 | sauce/ spread | mustard |
| 301 | sauce/ spread | oil |
| 302 | sauce/ spread | paste |
| 303 | sauce/ spread | pesto |
| 304 | sauce/ spread | salsa |
| 305 | sauce/ spread | sauce |
| 306 | sauce/ spread | soy |
| 307 | sauce/ spread | syrup |
| 308 | sauce/ spread | teriyaki |
| 309 | sauce/ spread | vinegar |
| 310 | snacks | chips |
| 311 | snacks | nachos |
| 312 | snacks | popcorn |
| 313 | snacks | snack |
| 314 | spice | chili |
| 315 | spice | cinnamon |
| 316 | spice | curry |
| 317 | spice | salt |
| 318 | spice | spice |
| 319 | spice | spicy |
| 320 | spice | sugar |
| 321 | spice | vanilla |
| 322 | sweets | brownie |
| 323 | sweets | cake |
| 324 | sweets | candy |
| 325 | sweets | caramel |
| 326 | sweets | chocolate |
| 327 | sweets | cobbler |
| 328 | sweets | cookie |
| 329 | sweets | cupcake |
| 330 | sweets | dessert |
| 331 | sweets | doughnut |
| 332 | sweets | flan |
| 333 | sweets | fudge |
| 334 | sweets | gum |
| 335 | sweets | halvah |
| 336 | sweets | honey |
| 337 | sweets | jelly |
| 338 | sweets | maple |
| 339 | sweets | marmalade |
| 340 | sweets | marshmallow |
| 341 | sweets | mousse |
| 342 | sweets | nougat |
| 343 | sweets | pancake |
| 344 | sweets | pudding |
| 345 | sweets | sorbet |
| 346 | sweets | strudel |
| 347 | sweets | toffee |
| 348 | sweets | tiramisu |
| 349 | vegetable | artichoke |
| 350 | vegetable | arugula |
| 351 | vegetable | asparagus |
| 352 | vegetable | aubergine |
| 353 | vegetable | basil |
| 354 | vegetable | beans |
| 355 | vegetable | beetroot |
| 356 | vegetable | bok choy |
| 357 | vegetable | broccoflower |
| 358 | vegetable | broccoli |
| 359 | vegetable | broccolini |
| 360 | vegetable | brussel sprouts |
| 361 | vegetable | cabbage |
| 362 | vegetable | carrot |
| 363 | vegetable | cassava |
| 364 | vegetable | cauliflower |
| 365 | vegetable | celeriac |
| 366 | vegetable | celery |
| 367 | vegetable | chamomile |
| 368 | vegetable | chard |
| 369 | vegetable | chipotle |
| 370 | vegetable | chive |
| 371 | vegetable | cilantro |
| 372 | vegetable | collard |
| 373 | vegetable | corn |
| 374 | vegetable | courgette |
| 375 | vegetable | cowpea |
| 376 | vegetable | cress |
| 377 | vegetable | cucumber |
| 378 | vegetable | eggplant |
| 379 | vegetable | endive |
| 380 | vegetable | fennel |
| 381 | vegetable | garlic |
| 382 | vegetable | ginger |
| 383 | vegetable | gumbo |
| 384 | vegetable | herbs |
| 385 | vegetable | hominy |
| 386 | vegetable | jalapeno |
| 387 | vegetable | kale |
| 388 | vegetable | kohlrabi |
| 389 | vegetable | leek |
| 390 | vegetable | lettuce |
| 391 | vegetable | microgreens |
| 392 | vegetable | mint |
| 393 | vegetable | mushroom |
| 394 | vegetable | onion |
| 395 | vegetable | parsley |
| 396 | vegetable | parsnip |
| 397 | vegetable | peas |
| 398 | vegetable | pepper |
| 399 | vegetable | pickles |
| 400 | vegetable | portobello |
| 401 | vegetable | potato |
| 402 | vegetable | puha |
| 403 | vegetable | pumpkin |
| 404 | vegetable | radish |
| 405 | vegetable | rhubarb |
| 406 | vegetable | seaweed |
| 407 | vegetable | shallot |
| 408 | vegetable | shitake |
| 409 | vegetable | silverbeet |
| 410 | vegetable | spinach |
| 411 | vegetable | squash |
| 412 | vegetable | swede |
| 413 | vegetable | taro |
| 414 | vegetable | tomato |
| 415 | vegetable | turnip |
| 416 | vegetable | vegetable |
| 417 | vegetable | watercress |
| 418 | vegetable | witloof |
| 419 | vegetable | yam |
| 420 | vegetable | zucchini |
