# Supplementary material for: Identifying Patients With Inflammatory Bowel Disease on Twitter and Learning From Their Personal Experience: Retrospective Cohort Study
Source: J Med Internet Res. 2022 Aug 2;24(8):e29186. doi: 10.2196/29186 (PMC9382547; doi:10.2196/29186)
Supplement: Multimedia Appendix 3 [file jmir_v24i8e29186_app3.docx]

## Multimedia Appendix 3

Table 1 and Table 2 show the LDA topics formatted on the training data for both Single-Instance (SI) and Multiple-Instance (MI) approaches, respectively. Highlighted in grey background are topic 11 of the SI approach and topics 17 and 9 of the MI approach, that were highly beneficial for the classification.

Table S1. LDA topics formatted on the training data for the Single-Instance approach.

| Topic | Top 20 words |
| --- | --- |
|  |  |
| 0 | link chronicillness ibd diagnosed women testing does spoonie affect expect expect testing women diagnosed affect women diagnosed expect chrons ibs low crohns link chrohns testing crohns |
| 1 | treat crohn link crohn ms alzheimer drug drug treat link treat alzheimer ms crohn alzheimer ms better individuals pelvic treat crohns phd use aspire function la |
| 2 | num ibd num num news link research ibdchat tweets week amp friends relationships live school people art anxiety day love anum |
| 3 | link writer melissa oconnell melissa oconnell gold stock stocks bitcoin girl stock ibd ibd almanactrader alexjones almanactrader hannity oprah suntimes nbc abc hannity alexjones foxbusiness |
| 4 | num crohn crohn disease disease years num years ve feel time healing tco ive https tco https diagnosed just vs work rates old |
| 5 | fail try known treatment provide crohns ulcerativecolitis steptherapy youve medication ibd health hcsm original required ulcerativecolitis providerprescribed known fail providerprescribed treatment health insurer ibd hcsm |
| 6 | artist music lover nature science ceo editor cat graduate enthusiast worshipper professor mother animal college writer proud group player sun |
| 7 | online colorectal pharma digital colorectal cancer magazine cancer diverticular disease diverticular business manufacturing free print bleeding occur cancer colitis including inflammatory disease angiodysplasia angiodysplasia owing colitis including |
| 8 | amp new link ibd crohns president kennedy patients did providers uc working partner look uc link ibd patients order research gastrointestinal numm |
| 9 | link colitis ulcerative ulcerative colitis crohn study team cause colitis link pain abdominal abdominal pain learn associated study link gene crohns colitis crohns parkinson wakeresearch |
| 10 | follow follow link sexy link rt disease looked rt follow looked sexy link looked sexy rt twitter expert expert panel panel qampa using gpibd submit holding feb num |
| 11 | amateur professional disability tweet advocate alum dad reddit junkie views host stocks cat employer enthusiast ft ma life fiction reading |
| 12 | ibd ecconum link num patients dr ecconum ibd ibd link ecconum link ecconum ecconum ibd ecconum clinical study ostomy care data treatment antitnf risk link link |
| 13 | anal today shingles colitis mad wish constipation pancreatitis crones sickle leakage sickle typhoid alopecia micro penis leakage chlamydia diphtheria anal celiac diphtheria chlamydia micro oab cow |
| 14 | effects crohn disease crohn disease marijuana new study study link complete remission new disease effects shows leads remission crohn leads complete marijuana leads complete remission effects new shows link |
| 15 | link crohns disease ibd crohns disease colitis amp life crohns colitis bowel know ulcerative ulcerative colitis love thank inflammatory need link link people support |
| 16 | tlrnum macrophages labour central multiple mainly drug project direct leader party lack alzheimers group intestinal cell cd patients appears patients link disease cd |
| 17 | come winds winds colitis et sea et intestinal come cells cdnum ends trms max qua leo et qua et max clubbing deformity deformity ends fingers result result crohns |
| 18 | im just health ibd colitis make crohns ibs help think issues nutrition like family microbiota fitness bloating crohns ibd ibs crohns gut |
| 19 | day map valentines valentines day crohns map vaccine daughter map vaccine happy valentines perfect walk late diagnosed uc link disability num year year day ibd cats catsgram whitecats |

Table S2. LDA topics formatted on the training data for the Multiple-Instance approach.

| Topic | Top 20 words |
| --- | --- |
|  |  |
| 0 | link breakingnews breakingnews trump news bbc link bbc news rt kim reddit korea donald north link link donald trump world link trump says north korea russian kim jong |
| 1 | link rt num num vedolizumab link link editorial pharmacokinetics rt num thanks people infliximab like link editorial num link ustekinumab new amp la just love ileal pouch |
| 2 | iheartfestival presale link link link rt head link just tickets like weve got craft beer new amp know make perfect register link link hey trump head im got |
| 3 | microbiome et et al al link al microbiota link rt gut ibd gut microbiota disease patients rectal bacterial microbial link link internal treatment oral intestinal |
| 4 | el en que la israel del link rt es link link por en el netanyahu para se link netanyahu link israel una mundial link el su |
| 5 | link rt num num rt num trump twitter link link just amp like new day know time live glutenfree im good news north make |
| 6 | la link rt link link en israel el que amp trump people im like al link israel think pray en el num num dont need |
| 7 | num num link rt new link link live world num link ibd amp israel rt num ostomy time army just link num im want people trump |
| 8 | ibd amp crohns new ulcerativecolitis health crohns ulcerativecolitis kennedy president fail colitis patients disease ibd patients ibd crohns ibdchat research coverage uc thank |
| 9 | announces new num link market link link global num num la data results june amp million link global num million report group annual news energy |
| 10 | link link link rt num num amp new just like num link thanks time people day rt num dont right today best know love retweet |
| 11 | link rt link link num num new tme cannabis live num link just like places workshops world love today time programme dukes robotic surgery know amp |
| 12 | pray christ promises gods num num link rt urgent prayer link urgent amp prayers fiance healing fiancé cannabis finances pray god new من link link |
| 13 | link rt num num amp rt num link link trump world just live new like dont israel years num link people good twitter love think |
| 14 | ibd stocks link rt gold link link oprah hannity nbc amp bonds abc oprah bitcoin link link gold ibdchat new israel patients stocks mixed crohns |
| 15 | link rt num num link link like just halo amp new love trailer know num link day rt num trump people israel good say man |
| 16 | fm kiss link link ariana link listen pete davidson pete davidson iheartradio link ariana free tickets ariana grande grande tickets tonyawards listen iheartfestival link nicki nicki link rt |
| 17 | link link link rt new amp world blue click like just blue link trump love num num dont people time num link science news twitter |
| 18 | לא link rt את זה num num על link link עם הוא rt num יש את זה אם trump say amp tens man just num link |
| 19 | num num link rt link link rt num amp new twitter trump people link num ibd news like world num link north make know love president |
