# Supplementary material for: Identifying Patients With Inflammatory Bowel Disease on Twitter and Learning From Their Personal Experience: Retrospective Cohort Study
Source: J Med Internet Res. 2022 Aug 2;24(8):e29186. doi: 10.2196/29186 (PMC9382547; doi:10.2196/29186)
Supplement: Multimedia Appendix 4 [file jmir_v24i8e29186_app4.pdf]

Multimedia Appendix 4

A network of connections between lifestyle-related words (lifestyles, in short) was built and visualized according to the statistics described in the section Analyzing Patients' Tweets. The different lifestyles were the nodes and an arc connected two lifestyles if they appeared in the same tweet. The more times they appeared together, the stronger the connection between the lifestyles was. Therefore, the resulted network was undirected and weighted by the number of times the lifestyles co-occurred. The purpose was to identify helpful lifestyles (frequently mentioned in a positive context) and lifestyles that are better to avoid (frequently mentioned in a negative context) and examine whether certain lifestyles tend to be implemented together.

The network was obtained using *Gephi* software for network analysis and visualization. Each node was colored on a scale from green to red based on the mean sentiment of the lifestyle it represented, green being very positive and red being very negative. The sizing of the nodes reflected the number of times the lifestyles were mentioned in the tweets database: the more times they appeared, the larger their nodes were. The thickness of each arc represented the number of times the two lifestyles it connected co-occurred: the thicker the arc, the more times the two lifestyles appeared together. To avoid obtaining an over-dense network, we only considered nodes of lifestyles mentioned at least five times in our database and included arcs between lifestyles that co-occurred at least four times. The process resulted in 144 lifestyles that are presented in the network.

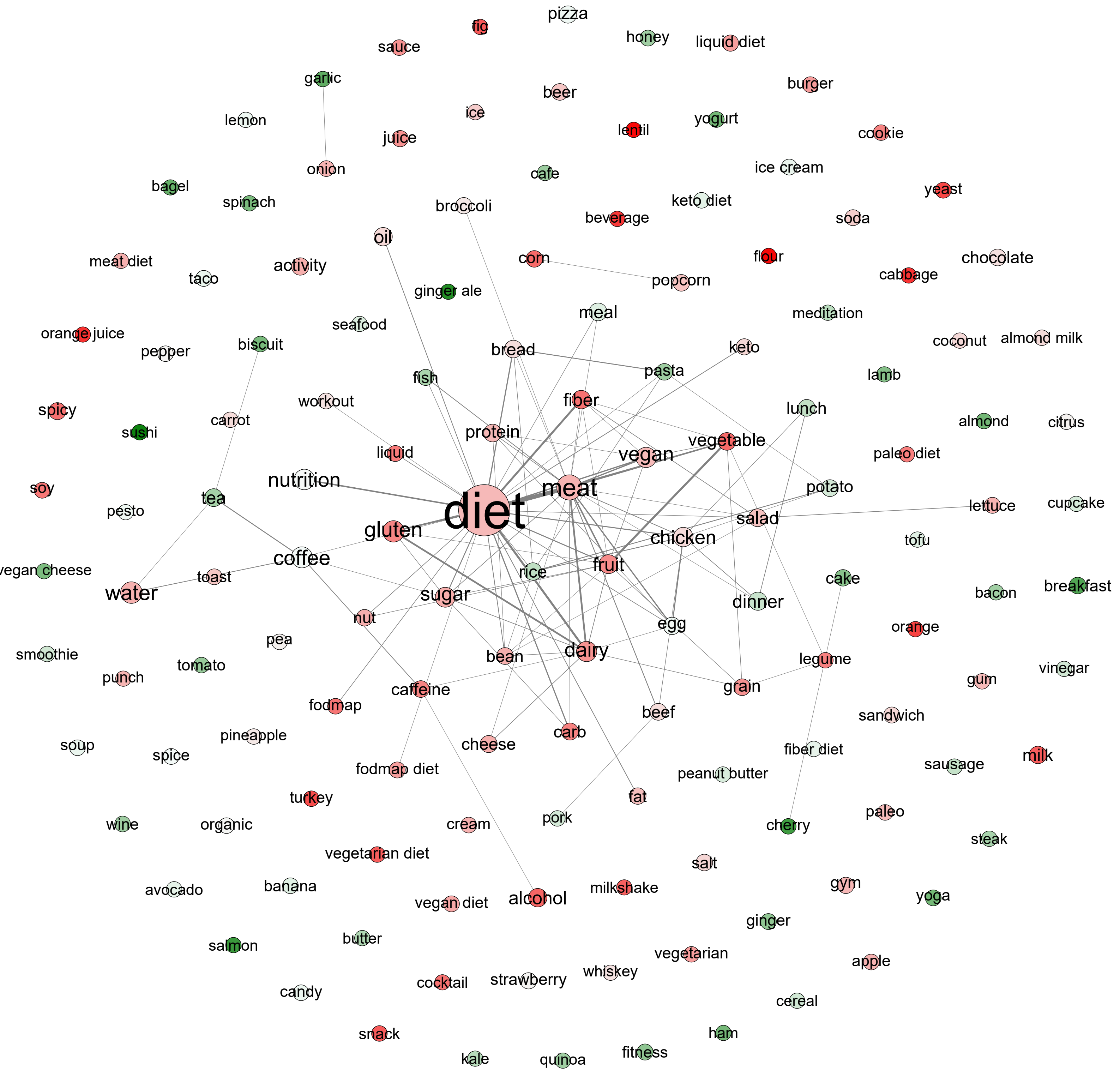
pizza
fig
honey
liquid diet
sauce
garlic
beer
burger
ice
lemon
lentil
yogurt
juice
cookie
onion
cafe
ice cream
bagel
yeast
spinach
broccoli
keto diet
beverage
soda
oil
meat diet
activity
corn
flour
chocolate
taco
popcorn
cabbage
ginger ale
meal
meditation
seafood
orange juice
biscuit
almond milk
pepper
bread
keto
coconut
pasta
lamb
fish
fiber
workout
spicy
lunch
sushi
carrot
almond
citrus
protein
vegetable
liquid
vegan
paleo diet
nutrition
soy
meat
potato
tea
diet
pesto
lettuce
cupcake
salad
gluten
chicken
tofu
coffee
vegan cheese
fruit
toast
rice
cake
water
breakfast
bacon
sugar
dinner
nut
egg
pea
orange
smoothie
dairy
legume
tomato
bean
vinegar
punch
gum
caffeine
grain
fodmap
beef
sandwich
pineapple
carb
soup
cheese
fiber diet
milk
spice
fodmap diet
sausage
peanut butter
turkey
fat
paleo
wine
organic
cream
pork
cherry
steak
vegetarian diet
salt
gym
avocado
banana
vegan diet
alcohol
milkshake
yoga
ginger
butter
salmon
vegetarian
apple
whiskey
cocktail
strawberry
candy
cereal
snack
ham
fitness
kale
quinoa
